# Metabolomic profiling of Ashwagandha (Withania somnifera): linking bioactive compounds to functional therapeutic roles

**DOI:** 10.3389/fmolb.2026.1796983

**Published:** 2026-08-05

**Authors:** Gaurav Tripathi, Vasundhra Bindal, Sanju Yadav, Manisha Yadav, Neha Sharma, Sushmita Pandey, Vipul Sharma, Nupur Sharma, Babu Mathew, Yash Magar, Shvetank Sharma, Shiv Kumar Sarin, Jaswinder Singh Maras

**Affiliations:** 1 Departments of Molecular and Cellular Medicine, Institute of Liver and Biliary Sciences, New Delhi, India; 2 Department of Hepatology, Institute of Liver and Biliary Sciences, New Delhi, India

**Keywords:** anti-inflammatory-, antioxidant, hepatoprotective action, metabolomics (LC-MS), withania somnifera, ashwagandha

## Abstract

**Background:**

*Withania somnifera* (Ashwagandha) is a widely used medicinal plant known for its neuroprotective, hepatoprotective, fertility-enhancing, and anti-inflammatory properties. Although it has a long history of use in traditional medicine, a detailed understanding of its phytochemical constituents and associated biological activities remains limited.

**Aim:**

To perform comprehensive metabolomic profiling of *Withania somnifera* and identify its major bioactive secondary metabolites, with a focus on compounds contributing to neuroprotection, liver health, reproductive support, and inflammation modulation.

**Methods:**

Root extracts of Withania somnifera (Ashwagandha) were subjected to methanol-based organic phase extraction followed by LC–MS/MS analysis, resulting in the characterization of 1,020 metabolites. Among these, 1,010 compounds were classified as putatively annotated (MSI Level 2/3 annotations) metabolites based on MS1 data, while 10 highly abundant metabolites were further confirmed by MS/MS fragmentation and assigned MSI Level 2 annotations. Relative abundance analysis was performed to determine the predominant phytochemical constituents of the extract. Key metabolites identified through this approach were subsequently correlated with the known pharmacological properties of Ashwagandha, including its immunomodulatory, anti-inflammatory, antioxidant, adaptogenic, and neuroprotective effects.

**Results:**

The analysis identified a diverse array of secondary metabolites, many present at low individual abundance ( <0.30% ). Notable compounds included the alkaloids Gentianaine ( 8.44% ) and Sauroxine ( 2.94% ), phenolics such as Mulberrofuran A ( 14.58% ) and Sulphuretin ( 9.61% ), and terpenoids including Pinocarvone and Ergosterol. (% of total annotated metabolites) These compounds were associated with anti-oxidative, neuro-regenerative, hepatoprotective, and anti-inflammatory effects. Additional metabolites, including Gomphrenin-I, Sitosterol, Pfaffic acid, and β -Carotene, were linked to reproductive health. Embelin and Kievitone were noted for their potential roles in enhancing oxidative stress responses. Despite the low abundance of individual compounds, the overall metabolite profile suggests substantial synergistic bioactivity.

**Conclusion:**

Metabolomic profiling of *Withania somnifera* reveals a rich composition of bioactive secondary metabolites with potential roles in neuroprotection, hepatoprotection, antioxidant defense, and reproductive support. These findings support its traditional use and highlight its potential for modern therapeutic applications in promoting stress resilience, vitality, and overall wellbeing.

## Graphical Abstract

**Figure:**
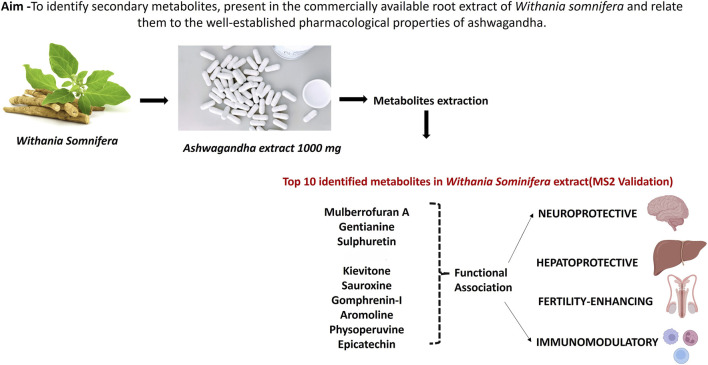
Summary of the study findings and key biological insights.

## Introduction

Withania somnifera, commonly known as Ashwagandha, is a cornerstone of Ayurveda medicine and has traditionally been used to promote vitality, longevity, and resilience to stress. Modern pharmacological investigations have begun to substantiate these traditional claims, revealing a broad spectrum of bioactivities attributable to its diverse phytochemical constituents (Mikulska et al., 2023).

Ashwagandha exhibits notable adaptogenic properties, helping to mitigate stress by modulating the hypothalamic-pituitary-adrenal (HPA) axis and reducing cortisol levels (Salve et al., 2019). Its neuroprotective effects are evidenced by improvements in cognitive function and memory, potentially through the upregulation of brain-derived neurotrophic factor (BDNF) and the attenuation of oxidative stress (Xing et al., 2022). Furthermore, Ashwagandha demonstrates anxiolytic and antidepressant activities, which may be mediated through modulation of GABAergic and serotonergic pathways (KrishnaRaju et al., 2023).

In the realm of immunology, Ashwagandha has demonstrated immunomodulatory effects, including the stimulation of lymphocyte proliferation, enhancement of natural killer (NK) cell activity, and increased macrophage phagocytic activity. Its anti-inflammatory properties are attributed to the suppression of pro-inflammatory cytokines and inhibition of the nuclear factor-kappa B (NF- κ B) signaling pathway (Alanazi and Elfaki, 2023).

Ashwagandha also contributes to metabolic health by improving insulin sensitivity and lipid profiles, thereby showing potential in the management of metabolic disorders. Its hepatoprotective effects have been observed through the reduction of liver enzyme levels and protection against hepatotoxic agents (Singh et al., 2011).

The herb’s influence extends to the gastrointestinal system, where it helps maintain gut integrity and modulate the microbiota, thereby contributing to overall digestive health. Notably, Ashwagandha has also been implicated in enhancing reproductive health, with studies reporting increased sperm count, motility, and testosterone levels in males, as well as improved sexual function in females (Roy et al., 2025).

Despite these promising findings, the specific secondary metabolites responsible for Ashwagandha’s well-known physiological effects remain poorly defined, and their underlying mechanisms are still incompletely understood. Advances in metabolomics now offer a comprehensive approach to elucidating the complex secondary metabolite profile of Ashwagandha and to linking it with its reported physiological activities.

In this study, we employed untargeted, high-resolution mass spectrometry-based metabolomics, coupled with secondary metabolite screening using the Plant Metabolome Database (PMDB), to profile the secondary metabolite landscape of Ashwagandha. By integrating functional annotation with the known physiological effects of Ashwagandha, we aimed to identify secondary metabolites that may contribute to hepatoprotective, neuroprotective, antioxidant, and spermatogenesis-enhancing activities.

## Methods

To ensure robust and comprehensive metabolomic profiling, approximately 1 mg of Withania somnifera (Ashwagandha) root extract per tablet (sourced from AIIA) was initially solubilized in 1 mL of RIPA buffer and subjected to high-energy probe sonication. RIPA buffer was used as an initial solubilisation and disruption medium to facilitate the release of phytochemicals from the dense herbal matrix. Because plant-derived materials contain cellulose-rich cell wall components that can hinder metabolite recovery, mechanical homogenization in the presence of RIPA buffer enhanced disruption of residual cellular structures and improved extraction efficiency. Following high-speed centrifugation, the supernatant was collected for metabolite extraction (Sharma et al., 2024; Sharma et al., 2022; Yadav et al., 2024; Challa et al., 2024; Mathew et al., 2024; Tripathi et al., 2021).

For metabolomic analysis, 200 µL of the recovered extract was mixed with 800 µL of chilled methanol (1:4, v/v) to precipitate proteins and enrich metabolites. The samples were incubated overnight at −20 °C and subsequently centrifuged at 13,000 rpm for 10 min. Overall; extraction was performed using a total solvent volume of 2 mL per 1 mg of extract, corresponding to an approximate sample-to-solvent ratio of 1:2000 (w/v). Both supernatant and pellet fractions were subjected to methanol-based organic extraction to maximize metabolite recovery.

Methanol precipitation preferentially enriched low-molecular-weight, semi-polar metabolites while minimizing interference from inactive formulation excipients, including binders, fillers, and coating materials. The resulting extracts were dried and reconstituted in 95% water and 5% acetonitrile (ACN) containing a mixture of internal and external standards. These standards were incorporated to assess extraction efficiency, monitor instrument performance, and validate analytical reproducibility across serial dilutions (Sharma et al., 2024; Sharma et al., 2022; Yadav et al., 2024; Challa et al., 2024; Mathew et al., 2024; Tripathi et al., 2021).

Metabolite separation was performed using reverse-phase chromatography on a Thermo Scientific™ C18 column (3 μm particle size, 2.1 mm × 100 mm) coupled with an ultra-high-performance liquid chromatography system linked to high-resolution Orbitrap mass spectrometry (UHPLC-HRMS). Mobile phase A was 0.1% formic acid, and mobile phase B was of 100% acetonitrile. A total of 15 µL samples were passed through the column into the heated Electrospray (HESI) source of a Q-Exactive mass spectrometer (Thermo Scientific, San Jose, CA) and was analysed both at positive and negative ionization modes. The spray voltage was 3.7 kV in positive and −3.1 kV in negative ionization mode. The heated capillary temperature was maintained at 360 °C, and the sheath/auxiliary gas flow was set to 15 and 10 (arbitrary units), respectively. All the samples were pooled together and spiked with internal standards (Dinose b: 1 mg/mL; MCPA: 1 mg/mL; Dimetrazole: 1 mg/mL) and external standards (Cholesterol: 0.1 mg/mL; Colchicine: 4 mg/mL; Imipramine: 2 mg/mL; Roxithromycin: 2 mg/mL; Amiloride: 1 mg/mL; Atropine:2 mg/mL) and were analysed in serial dilutions of 1:1,1:2,1:4 and 1:8 for determining analytical reproducibility. The MS/MS mode isolation width was set to m/z 1.5, the normalized collision energy was 32%, and the mass resolution was set at 17,500 FWHM at m/z 200.

Only ions demonstrating strong reproducibility (CV <30% across technical replicates) and robust correlation (*r* ^2^ > 0.7) were retained for annotation and downstream analysis. Technical replicates confirmed high analytical stability, with all selected features meeting the predefined precision threshold of CV <30%.

Annotation against the Plant Metabolite Database (PMDB v2.4; http://www.pmdb.org.cn/home) using Compound Discoverer 3.0 identified a total of 1,020 metabolites. Metabolite annotations were assigned based on high-resolution accurate mass matching with a stringent mass error threshold of <1 ppm. Among the identified compounds, 1,010 were classified as putatively annotated metabolites (MSI Level 2/3), while the 10 most abundant metabolites were further validated by MS/MS fragmentation and assigned MSI Level 2 annotations.

The identified metabolites encompassed a diverse range of phytochemical classes, including alkaloids, phenolics, terpenoids, and six major categories of plant hormones, highlighting the extensive chemical diversity of Withania somnifera. This comprehensive, high-resolution metabolomic approach provides valuable insights into the phytochemical architecture of Ashwagandha and establishes a foundation for linking its metabolome with its documented pharmacological properties.

**FIGURE 1:**
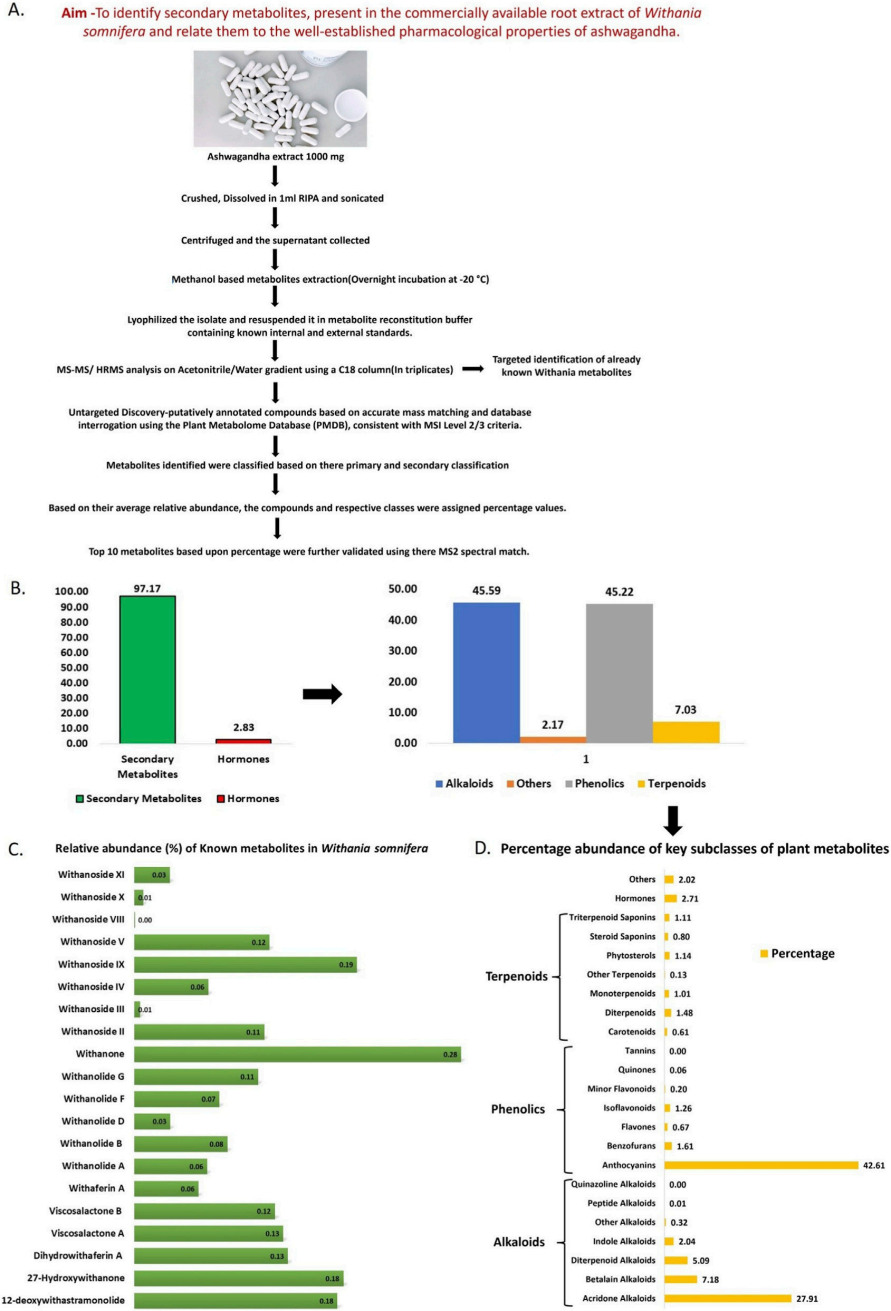
Workflow for metabolite extraction, identification, and compositional analysis of commercial Withania somnifera root extract. **(A)** Schematic overview of the experimental workflow used to identify secondary metabolites from the commercially available Withania somnifera root extract. **(B)** Percentage of secondary metabolites and hormones in the analyzed extract, along with class-wise distribution of the detected compounds into alkaloids, phenolics, terpenoids, and other metabolites. **(C)** Percentage abundance of known metabolites detected in Withania somnifera, including withanolides, Withanolides, and related specialized metabolites. **(D)** Percentage abundance of major metabolite subclasses identified in the plant extract, grouped into alkaloids, phenolics, terpenoids, hormones, and other compounds and subclasses.

To determine the relative phytochemical composition, all annotated metabolites were first compiled and their peak intensities summed. The percentage abundance of each metabolite was then calculated relative to the total cumulative intensity of all annotated compounds. This approach enabled quantitative assessment of the contribution of individual metabolites to the overall metabolomic profile and facilitated identification of the predominant bioactive compounds. Microsoft Excel was used to calculate the mean values and standard deviations (SD) for compound abundance data obtained from biological replicates (Figure 1A).

## Results

### Distribution of withanolides and other known compounds in Withania somnifera based on percentage abundance

The metabolic profiling of Withania somnifera reveals a diverse range of withanolides and related compounds present in varying proportions. Among these, withanone shows the highest percentage abundance at 0.28%, followed by withanoside IX at 0.19%, and both 12-deoxywithastramonolide and 27-hydroxywithanone at 0.18% each. Other notable compounds include dihydrowithaferin A and viscosalactone A, each contributing 0.13%, while viscostalactone B and withanoside V are present at 0.12%. Withanolide G and withanoside II show moderate abundance at 0.11%, whereas withanolide F, Withanolide B, range between 0.06% and 0.08% (% of total annotated metabolites). Compounds such as withaferin A, withanolide A, and withanoside IV are detected at 0.06%, while withanolide D, withanoside XI, and withanoside III are among the least abundant, each contributing below 0.03%. Notably, withanoside VIII shows no detectable abundance in the profile. This distribution highlights the chemical complexity and variability of bioactive withanolides in Withania somnifera (Figure 1C).

### Major phytochemical classes in Ashwagandha extract (percentage distribution)

The metabolomic profiling of Withania somnifera (Ashwagandha) revealed a diverse and functionally enriched phytochemical composition, dominated by phenolic and alkaloid subclasses. Anthocyanins emerged as the most abundant class, accounting for 42.61% of the total phytochemical content, followed by acridone alkaloids (27.91%), betaine alkaloids (7.18%), and diterpenoid alkaloids (5.09%). Other notable contributors included indole alkaloids (2.04%), plant-derived hormones (2.71%), isoflavonoids (1.26%), diterpenoids (1.48%), triterpenoid saponins (1.11%), phytosterols (1.14%), and monoterpenoids (1.01%). Minor constituents such as quinazoline alkaloids, peptide alkaloids, quinones, and tannins were also detected, reflecting the chemotypic complexity of the extract. This broad-spectrum phytochemical distribution underscores Ashwagandha’s potential for multifaceted therapeutic activity, including neuroprotection, immunomodulation, metabolic regulation, and redox homeostasis, aligning with its traditional use in adaptogenic and rejuvenative medicine (Figures 1B–D).

**FIGURE 2:**
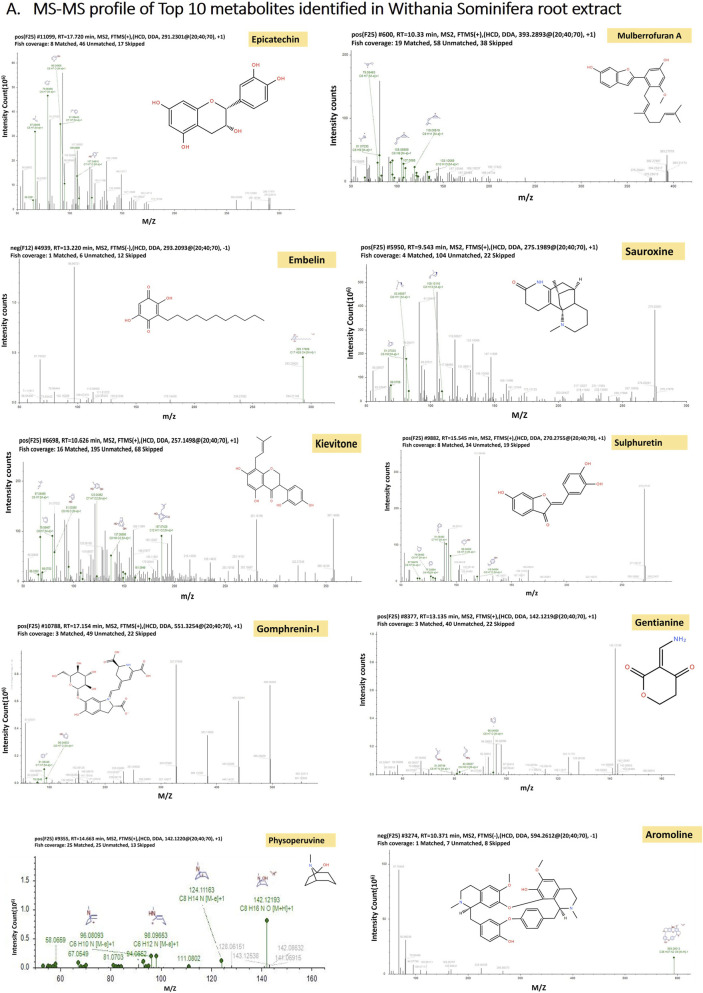
MS-MS profile of Top 10 metabolites identified in Withania Somnifera root extract.

The top compounds identified in Ashwagandha, along with their percentage abundance (% of total annotated metabolites)., include Mulberrofuran A (14.58%), Gentianaine (8.44%), and Sulphuretin (4.43%). Other notable compounds include Embelin (4.10%), Kievitone (3.05%), and Sauroxine (2.95%). Gomphrenin-I accounts for 2.89%, while Aromoline is present at 2.43%. Physoperuvine has a percentage abundance of 2.29%, and Epicatechin is found at 1.96%. Coronaridine and Afzelechin-(4α→8)-afzelechin are present at 1.95% and 1.73%, respectively. Additionally, Hildecarpin (1.70%), Poncirin (1.62%), and Muscapurpurin (1.42%) were observed. Other compounds include Rescinnamine (1.38%), Cadiamine (1.18%), Morindone (1.14%), Pseudobaptigenin (1.13%), and Gibberellin A15 (1.06%) (Table 1). These compounds were further validated at MS2 levels (Figure 2).

**TABLE 1:** Table presenting the top 10 key metabolites identified in Withania somnifera using mass spectrometry, including m/z values, retention times, and related parameters. (% compared to total compounds).

Metabolite	PMDB ID	Calc. MW	RT [min]	M/Z	Formula	Percentage abundance	MSI identifcation
Mulberrofuran A	PMDB00542	392.2907	12.375	391.2835	C24 H40 O4	14.58224358	MS2 validated MSI level 2
Gentianine	PMDB00318	141.1145	13.111	142.1218	C6 H7 N O3	8.4449061	MS2 validated MSI level 2
Sulphuretin	PMDB00531	270.1446	13.684	271.1519	C10 H18 N6 O3	4.430593245	MS2 validated MSI level 2
Embelin	PMDB00821	294.1848	14.871	293.1775	C16 H27 N2 O P	4.095842766	MS2 validated MSI level 2
Kievitone	PMDB00879	356.2689	16.625	357.2762	C19 H36 N2 O4	3.047951978	MS2 validated MSI level 2
Sauroxine	PMDB00301	274.1939	10.569	590.4217	C18 H26 O2	2.946995637	MS2 validated MSI level 2
Gomphrenin-I	PMDB00062	550.344	14.293	551.3513	C30 H52 N2 O3 P2	2.889142499	MS2 validated MSI level 2
Aromoline	PMDB00495	594.3695	18.631	595.3768	C40 H50 O4	2.431618825	MS2 validated MSI level 2
Physoperuvine	PMDB00466	141.1146	1.074	142.1219	C8 H15 N O	2.29419267	MS2 validated MSI level 2
Epicatechin	PMDB00566	290.2223	15.375	291.2296	C15 H26 N6	1.958538555	MS2 validated MSI level 2

### Key alkaloids and subclasses in Ashwagandha

Out of the total secondary metabolites, which account for 97.17% of the extract, alkaloids represent 44.30%. The alkaloid profile is primarily dominated by acridone alkaloids, which contribute 27.91%. Betalain alkaloids follow at 7.18%, while diterpenoid alkaloids are present at 5.09%. Indole alkaloids account for 2.04%, and other alkaloids make up 0.32%. Peptide alkaloids are present only in trace amounts at 0.01%, and quinazoline alkaloids are the least abundant, representing just 0.002% (Figures 1B–D).

If the total alkaloid pool is normalized to 100%, the key alkaloids contribute the following proportions: Gentianaine is the most abundant, representing 19.98% of total alkaloids. Sauroxine 6.97% respectively. Aromoline and Physoperuvine are present at 5.75% and 5.43%, respectively, while Coronaridine contributes 4.60%. Other notable alkaloids include Muscapurpurin (3.36%), Rescinnamine (3.26%), and Cadiamine (2.79%). Betanin, Vasicinone, and Portulacaxanthin II account for 1.75%, 1.71%, and 1.71% of the total, respectively. Minor alkaloids such as Miraxanthin-II (1.45%), Akuammicine (1.26%), Vulgaxanthin-I (1.15%), and Ergosine (1.03%) contribute smaller proportions. Ergonovine, Akuammidine, Calebassone, and Garryine are present in the lowest amounts, each contributing less than 1% (Table 3).

### Key phenolics and subclasses in Ashwagandha

Out of the total phenolic compounds, anthocyanins make up the largest proportion at 42.61% . Other phenolic subclasses include benzofurans ( 1.61% ), isoflavonoids ( 1.26% ), and flavones ( 0.67% ). Minor flavonoids contribute 0.20% , while quinones and tannins are present in very small amounts, contributing 0.06% and 0.004% , respectively. Overall, phenolics account for 43.94% of the extract.

When the total phenolic fraction is normalized to 100% , the key phenolics contribute the following proportions: Mulberrofuran A is the most abundant, representing 31.63% of total phenolics. Sulphuretin and Embelin follow, accounting for 9.61% and 8.88% , respectively. Kievitone contributes 6.61% , while Epicatechin and Afzelechin-( 4α→8 )-afzelechin are present at 4.25% and 3.74% . Other notable phenolics include Hildecarpin ( 3.68% ), Poncirin ( 3.51% ), and Morindone ( 2.47% ). Pseudobaptigenin contributes 2.45% , Barbaloin 1.88% , and Isodiospyrin 1.57% . Irisolidone, Rapanone, and Catechin account for 1.40% , 1.15% , and 1.08% , respectively. Minor phenolics such as Diospyrin ( 0.93% ), Norobtusifolin ( 0.90% ), Dihydromyricetin ( 0.84% ), β -Lapachone ( 0.79% ), and Lawsone ( 0.55% ) contribute smaller amounts to the overall phenolic pool (Table 2).

**TABLE 2:** Percentage distribution of key Phenolics detected in Withania somnifera using LC-MS/MS. (% compared to total Phenolics).

Key phenolics	Percentage abundance	Probable biological activity	Relevance	MSI identifcation
Mulberrofuran A	31.63	Antiviral, antioxidant, anti-inflammatory	Brain health (neuroinflammation)	MS2 Validated MSI level 2
Sulphuretin	9.61	Anti-inflammatory, neuroprotective, antioxidant	Brain health	MS2 Validated MSI level 2
Embelin	8.88	Antioxidant, pro-apoptotic (cancer), antifertility	Negative effect on spermatogenesis	MS2 Validated MSI level 2
Kievitone	6.61	Antioxidant, anti-inflammatory	General health	MS2 Validated MSI level 2
Epicatechin	4.25	Improves blood flow, neurogenesis, antioxidant	Brain and reproductive health	MS2 Validated MSI level 2
Afzelechin−(4alpha−>8)-afzelechin	3.74	Antioxidant, neuroprotective (flavan-3−ol dimer)	Brain health	MS2 Validated MSI level 3
Hildecarpin	3.68	NA	NA	MS2 Validated MSI level 3
Poncirin	3.51	Anti-inflammatory, hepatoprotective, neuroprotective	Brain and thyroid (indirect)	MS2 Validated MSI level 3
Morindone	2.47	Anticancer, antioxidant	General health	MS2 Validated MSI level 3
Pseudobaptigenin	2.45	Estrogenic/phytoestrogen activity	Hormonal regulation	MS2 Validated MSI level 3
Barbaloin	1.88	Anti-inflammatory, gut health, hepatoprotective	General/thyroid via liver support	MS2 Validated MSI level 3
Isodiospyrin	1.57	Phytoestrogen, neuroprotective, antioxidant	Brain and hormonal balance	MS2 Validated MSI level 3
Irisolidone	1.4	Antimicrobial, potential anticancer	General	MS2 Validated MSI level 3
Rapanone	1.15	Cytotoxic, antioxidant	General	MS2 Validated MSI level 3
Catechin	1.08	Enhances cognition, antioxidant, boosts testosterone	Brain and spermatogenesis	MS2 Validated MSI level 3
Diospyrin	0.93	Antimicrobial, limited CNS activity known	General	MS2 Validated MSI level 3
Norobtusifolin	0.9	NA	NA	MS2 Validated MSI level 3
Dihydromyricetin	0.84	Neuroprotective, anti-alcohol toxicity, antioxidant	Brain health	MS2 Validated MSI level 3
beta−Lapachone	0.79	Anticancer, anti-inflammatory, potential neuroprotection	Brain health	MS2 Validated MSI level 3
Lawsone	0.55	Antioxidant, antifungal, may affect hormonal balance	General, possible thyroid influence	MS2 Validated MSI level 3

**TABLE 3:** Percentage distribution of key alkaloids detected in Withania somnifera using LC-MS/MS. (% compared to total Alkaloids).

Key phenolics	Percentage abundance	Probable biological activity	Relevance	MSI identifcation
Gentianaine	19.98	Antioxidant, anti-inflammatory	Brain health	MS2 Validated MSI level 2
Sauroxine	6.97	NA	NA	MS2 Validated MSI level 2
Aromoline	5.75	NA	NA	MS2 Validated MSI level 2
Physoperuvine	5.43	Anticholinesterase, neuroprotective	Brain health	MS2 Validated MSI level 2
Coronaridine	4.6	Anti-addictive, acetylcholinesterase inhibitor	Brain health	MS2 Validated MSI level 3
Muscarpurin	3.36	Antioxidant (betalain type)	Brain and general metabolism	MS2 Validated MSI level 3
Rescinnamine	3.26	Antihypertensive (RAAS pathway modulator)	Brain health (vascular-related)	MS2 Validated MSI level 3
Cadiamine	2.79	NA	NA	MS2 Validated MSI level 3
Betanin	1.75	Strong antioxidant, hepatoprotective	Thyroid (indirect support)	MS2 Validated MSI level 3
Portulacaxanthin II	1.71	Antioxidant, neuroprotective (betalain pigment)	Brain health	MS2 Validated MSI level 3
Vasicinone	1.71	Anti-inflammatory, uterotonic, mucolytic	Potential hormonal support	MS2 Validated MSI level 3
Miraxanthin−II	1.45	Antioxidant, anti-inflammatory	General health	MS2 Validated MSI level 3
Akuammicine	1.26	Analgesic, neuroactive (opioid receptor modulator)	Brain health	MS2 Validated MSI level 3
Vulgaxanthin−I	1.15	Antioxidant (betalain)	Brain and metabolic health	MS2 Validated MSI level 3
Ergosine	1.03	Dopaminergic, oxytocic activity	Brain and reproductive systems	MS2 Validated MSI level 3
Ergonovine	0.93	Uterotonic, oxytocic (ergot alkaloid)	Reproductive system	MS2 Validated MSI level 3
Akuammidine	0.78	Neuroactive, analgesic, anti-inflammatory	Brain health	MS2 Validated MSI level 3
Calebassone	0.78	NA	NA	MS2 Validated MSI level 3
Garryine	0.75	NA	NA	MS2 Validated MSI level 3

### Key terpenoids and subclasses in Ashwagandha

When the total terpenoids fraction is normalized to 100%. Among the terpenoids, diterpenoids are the most abundant, accounting for 1.48% . This is followed by phytosterols at 1.14% and triterpenoid saponins at 1.11% . Monoterpenoids contribute 1.01% , while steroid saponins make up 0.80% . Carotenoids represent 0.61% , and other terpenoids are present in the smallest proportion at 0.13% . Overall, terpenoids account for 6.83% of the extract (Figure 1B).

The key terpenoids contribute the following proportions: Pinocarvone is the most abundant, making up 9.19% of total terpenoids. Ergosterol follows at 7.06% , while 6-Acetylpicropolin contributes 6.09% . Sitosterol and Diosgenin account for 5.29% and 5.00% , respectively. Alectrol and Gitonin contribute 4.74% and 3.68% , respectively. Pfaffic acid ( 3.65% ) and Aphidicolin ( 2.92% ) are also notable contributors. Other terpenoids include Quillaic acid ( 2.90% ), β -Carotene ( 2.55% ), and Ilexolide A ( 2.44% ). Chrysanthemic acid ( 2.27% ), Cafestol ( 2.09% ), and Ziziphin ( 1.96% ) follow. Withanolide D, Olaxoside, and Miroestrol are present at 1.92% , 1.91% , and 1.91% , respectively. Casbene and Echinenone contribute the smallest amounts, each at 1.70% of the total terpenoid concentration (Table 4).

**TABLE 4:** Percentage distribution of key terpenoids detected in Withania somnifera using LC-MS/MS**.** (% compared to total Terpenoids).

Key terpenoids	Percentage abundance	Probable biological activity	Relevance	MSI identifcation
Pinocarvone	9.19	Antioxidant, antimicrobial	Brain (limited evidence)	MS2 Validated MSI level 3
Ergosterol	7.06	Precursor of vitamin D2, anti-inflammatory	Brain, potential hormonal modulation	MS2 Validated MSI level 3
6-Acetylpicropolin	6.09	NA	NA	MS2 Validated MSI level 3
Sitosterol	5.29	Cholesterol-lowering, enhances sperm parameters	Spermatogenesis	MS2 Validated MSI level 3
Diosgenin	5.00	Precursor to steroid hormones, improves cognition	Spermatogenesis, brain, thyroid (indirect)	MS2 Validated MSI level 3
Alectrol	4.74	NA	NA	MS2 Validated MSI level 3
Gitonin	3.68	Cardiac glycoside; possible anti-inflammatory	Potential hormonal effects	MS2 Validated MSI level 3
Pfaffic acid	3.65	Anti-fatigue, enhances libido and fertility	Spermatogenesis	MS2 Validated MSI level 3
Aphidicolin	2.92	DNA polymerase inhibitor, anticancer	General (not for routine supplementation)	MS2 Validated MSI level 3
Quillaic acid	2.90	Saponin, immunostimulatory	General health, immune-brain link	MS2 Validated MSI level 3
beta−Carotene	2.55	Antioxidant, vitamin A precursor, neuroprotective	Brain and sperm health	MS2 Validated MSI level 3
Ilexolide A	2.44	NA	NA	MS2 Validated MSI level 3
Chrysanthemic acid	2.27	Insecticidal, antioxidant	General (indirect brain antioxidant role)	MS2 Validated MSI level 3
Cafestol	2.09	Modulates bi`le acid metabolism, antioxidant	Liver-thyroid axis (indirect)	MS2 Validated MSI level 3
Ziziphin	1.96	Sweet taste modifier; triterpenoid	General (no direct reproductive role)	MS2 Validated MSI level 3
Withanolide D	1.92	Neuroprotective, anti-inflammatory, adaptogenic	Brain, thyroid, and potentially sperm	MS2 Validated MSI level 3
Olaxoside	1.91	NA	NA	MS2 Validated MSI level 3
Miroestrol	1.91	Phytoestrogen, hormonal modulation	Reproductive hormone regulation	MS2 Validated MSI level 3
Casbene	1.70	Tumor-promoting diterpene (at high doses)	Not favorable	MS2 Validated MSI level 3
Echinenone	1.70	Carotenoid, antioxidant, immunomodulatory	Brain and sperm health	MS2 Validated MSI level 3

### Physiological effect of ashwagandha metabolites

Withania somnifera (Ashwagandha) exerts a wide range of physiological effects in the human body, largely due to its rich composition of bioactive compounds with diverse pharmacological properties. Key constituents such as Mulberrofuran A, Gentianaine, and Sulphuretin, which are present in relatively high abundance, contribute significantly to Ashwagandha’s anti-inflammatory, antioxidant, antiviral, and anticancer activities (Kim et al., 2022; Mirzaee et al., 2017; Song et al., 2010). For instance, Mulberrofuran A ( 14.58% ) demonstrates strong anti-inflammatory and antiviral effects, supporting immune modulation and protection against infections. Gentianaine ( 8.44% ) offers neuroprotective and antibacterial benefits, helping safeguard the nervous system while combating microbial pathogens. Sulphuretin ( 4.43% ) further enhances antioxidant defenses and exhibits anti-inflammatory and anticancer properties, promoting cellular health and reducing oxidative stress.

Other compounds, such as Embelin and Kievitone, also contribute to Ashwagandha’s antioxidant and antimicrobial effects, supporting overall immune function and potentially inhibiting tumor growth. Gomphrenin-I and Muscapurpurin provide hepatoprotective effects, helping protect liver function by reducing inflammation and oxidative damage. The presence of Epicatechin and afzelechin derivatives adds cardioprotective, antidiabetic, and anti-inflammatory benefits, which are crucial for metabolic and cardiovascular health (Mirzaee et al., 2017; Abdelsalam et al., 2023; Hid et al., 2022; Kamble et al., 2020).

Additionally, compounds such as Sauroxine and Physoperuvine suggest potential central nervous system (CNS) stimulatory and anticholinergic activities, indicating Ashwagandha’s possible role in modulating cognitive function and neurological health. Other constituents, including Poncirin and Pseudobaptigenin, exhibit anti-inflammatory, antiallergic, and estrogenic activities, are reflecting the adaptogen’s broad hormonal and immunomodulatory effects (KrishnaRaju et al., 2023; Vallejo et al., 2009).

Together, these bioactive molecules may act synergistically to influence multiple physiological pathways, reducing inflammation, protecting vital organs, enhancing neurological function, and supporting cardiovascular and metabolic health. Collectively, these effects underpin Ashwagandha’s reputation as a powerful adaptogenic and therapeutic herb.

## Discussion

The diverse and functionally enriched phytochemical profile of *Withania somnifera* (Ashwagandha) underpins its wide-ranging therapeutic activities, including neuroprotection, hepatoprotection, spermatogenesis support, and anti-inflammatory effects. Metabolomic profiling reveals a complex array of secondary metabolites, dominated by alkaloids and phenolic compounds, which together provide a strong basis for its traditional and emerging medical applications. In this study, we examined the percentage distribution of major secondary metabolites and their association with multifaceted health benefits, including neuroprotection, hepatoprotection, spermatogenesis stimulation, and anti-inflammatory activity (Mikulska et al., 2023; Salve et al., 2019; Xing et al., 2022; KrishnaRaju et al., 2023; Alanazi and Elfaki, 2023; Singh et al., 2011).

The neuroprotective properties of Ashwagandha are primarily attributed to its rich alkaloid and phenolic content, particularly compounds such as Gentianaine, Mulberrofuran A, and Sulphuretin, which play pivotal roles in protecting the brain from oxidative stress and neuroinflammation. The alkaloid class, with Gentianaine as the most abundant compound ( 19.98% of total alkaloids), has shown strong antioxidant potential, helping protect neurons from free radical-induced damage (Wang et al., 2021). Mulberrofuran A, the most abundant phenolic compound ( 31.63% of total phenolics), is also known for its neuroprotective effects, supporting neuronal growth and regeneration. (Kim et al., 2022). These compounds, along with Sulphuretin ( 9.61% of total phenolics), promote cellular health and may help prevent or delay the onset of neurodegenerative diseases such as Alzheimer’s and Parkinson’s. Furthermore, Embelin and Kievitone, which together account for over 10% of the phenolic content, exhibit neuroprotective properties by modulating oxidative stress pathways and promoting neuronal cell survival (Abdelsalam et al., 2023; Kamble et al., 2020). The presence of Aromoline and Sauroxine within the alkaloid fraction, both of which have been implicated in neuroprotective mechanisms, further enhances Ashwagandha’s potential in the management of neurodegenerative conditions (Vallejo et al., 2009; Hostalkova et al., 2019).

Ashwagandha also demonstrates considerable hepatoprotective properties, driven largely by its diverse alkaloid and phenolic content. Acridone alkaloids, which contribute 27.91% of total alkaloids, are known to exert protective effects against liver toxicity by modulating oxidative stress and inflammation. Notably, Gentianaine and Sauroxine ( 6.97% and 6.83% of alkaloids, respectively) have been found to support liver function by reducing hepatocellular damage (Mirzaee et al., 2017; Vallejo et al., 2009). Mulberrofuran A and Sulphuretin, key phenolic compounds, also play significant roles in liver protection by reducing lipid peroxidation and enhancing antioxidant defenses, thereby mitigating liver damage associated with chronic conditions such as alcoholic liver disease and non-alcoholic fatty liver disease (NAFLD) (Kim et al., 2022; Song et al., 2010). Additionally, Pinocarvone (Mohamed and Younis, 2022) and Ergosterol, two important terpenoids, have been shown to exhibit hepatoprotective properties through their anti-inflammatory and antioxidative effects, helping to prevent liver fibrosis and improve detoxification pathways. Together with Ashwagandha’s overall rich phytochemical composition, these compounds highlight its potential as a natural adjunct in the management of liver diseases (Merdivan and Lindequist, 2017; Miyaji et al., 2016).

The role of Ashwagandha in promoting male reproductive health is another area of considerable interest. Gomphrenin-I (2.88% of total) and Sauroxine are key contributors to testosterone production and spermatogenesis. Gomphrenin-I has been reported to significantly improve sperm count, motility, and morphology, making it a valuable compound in the management of male infertility (Vallejo et al., 2009; Lin et al., 2010). These compounds work synergistically to improve the health of the testes, increase sperm viability, and potentially boost overall male fertility.

Ashwagandha’s anti-inflammatory activity is one of its most significant therapeutic attributes. Its phytochemicals, particularly Sulphuretin, Mulberrofuran A, and Betanin, have demonstrated strong anti-inflammatory effects by modulating pro-inflammatory cytokines such as TNF-α, IL-1β, and IL-6. Sulphuretin ( 9.61% of total phenolics) is especially noteworthy for its ability to suppress inflammatory pathways in both the brain and peripheral tissues, offering protection against conditions such as rheumatoid arthritis and chronic inflammation (Kim et al., 2022; Silva et al., 2022).

Additionally, Pinocarvone and Ergosterol, key terpenoid compounds, further enhance Ashwagandha’s anti-inflammatory profile by reducing inflammatory cytokine release and promoting immune modulation. Together, these compounds may help alleviate symptoms associated with chronic inflammatory diseases and reduce the risk of systemic inflammation-related conditions such as cardiovascular disease, diabetes, and neurodegenerative disorders (Merdivan and Lindequist, 2017; Miyaji et al., 2016).

In conclusion, the remarkable phytochemical diversity of Withania somnifera (Ashwagandha) underpins its promise as a multifaceted therapeutic agent. Its neuroprotective, hepatoprotective, spermatogenesis-enhancing, and anti-inflammatory effects appear to arise from the synergistic actions of bioactive compounds such as Gentianaine, Mulberrofuran A, and Sulphuretin. As understanding of these compounds and their molecular mechanisms continues to expand, Ashwagandha’s potential as an integrative intervention for chronic conditions ranging from neurodegenerative diseases to infertility and liver disorders becomes increasingly apparent.

The metabolite-identification strategy used in this study, based on Plant Metabolome Database annotation, offers a useful framework for analyzing herbal products and characterizing key secondary metabolites that may contribute to the pathophysiological effects of traditional Ayurvedic formulations and drugs.

## Lacunae and future scope of the study

The present study investigated a commercially available Withania somnifera (Ashwagandha) root extract rather than the whole plant. Consequently, the phytochemical profiles of different plant tissues, including roots, leaves, stems, berries, and seeds, were not assessed. Comparative metabolomic analyses across various plant parts, cultivars, and formulations would provide a more comprehensive understanding of the phytochemical diversity of W. somnifera and its therapeutic relevance in Ayurvedic medicine.

Metabolite identification was primarily based on accurate mass matching and database-assisted annotation using the Plant Metabolome Database (PMDB v2.4). Although 1,020 metabolites were annotated, only the ten most abundant compounds were further validated through MS/MS spectral matching and assigned MSI Level 2 confidence. Therefore, the majority of metabolites remain putatively annotated and require confirmation using authentic reference standards, targeted LC–MS/MS, retention time matching, and complementary structural characterization approaches such as NMR spectroscopy.

A major limitation of this study was the limited availability of authentic standards for many plant-derived metabolites, which precluded absolute quantification and comprehensive biological validation. Furthermore, differences in ionization efficiency among metabolites may have influenced relative abundance estimates. Future investigations should incorporate targeted quantitative metabolomics, isotopically labeled standards, and ionization-efficiency correction strategies to improve metabolite quantification and annotation confidence.

Another important limitation is that the biological activities attributed to the identified metabolites were not experimentally evaluated in the present study. Instead, functional interpretations were inferred from published literature, database annotations, and previously reported pharmacological properties. Future studies should therefore validate the biological effects of key metabolites through *in vitro*, *ex vivo*, and *in vivo* experiments, including investigations of their antioxidant, anti-inflammatory, immunomodulatory, neuroprotective, adaptogenic, and metabolic activities.

Finally, integration of metabolomics with transcriptomics, proteomics, network pharmacology, and systems biology approaches may provide deeper mechanistic insights into the molecular basis of Ashwagandha’s therapeutic effects and facilitate the identification of bioactive compounds suitable for standardization, quality control, drug discovery, and evidence-based clinical applications.

## Data Availability

The data presented in the study are deposited in the Figshare repository, accession number https://doi.org/10.6084/m9.figshare.33102755.
